# Proteomic mapping and optogenetic manipulation of membrane contact sites

**DOI:** 10.1042/BCJ20220382

**Published:** 2022-09-16

**Authors:** Gang Lin, Wenyi Shi, Ningxia Zhang, Yi-Tsang Lee, Youjun Wang, Ji Jing

**Affiliations:** 1 The Cancer Hospital of the University of Chinese Academy of Sciences (Zhejiang Cancer Hospital), Institute of Basic Medicine and Cancer (IBMC), Chinese Academy of Sciences, Hangzhou, Zhejiang 310022, China; 2 School of Molecular Medicine, Hangzhou Institute for Advanced Study, University of Chinese Academy of Sciences (UCAS), Hangzhou 310024, China; 3 Laboratory of Cancer Biology, Department of Medical Oncology, Institute of Clinical Science, Sir Run Run Shaw Hospital, College of Medicine, Zhejiang University, Hangzhou, China; 4 Center for Translational Cancer Research, Institute of Biosciences and Technology Department of Translational Medical Sciences, College of Medicine, Texas A&M University, Houston, TX 77030, U.S.A.; 5 Beijing Key Laboratory of Gene Resource and Molecular Development, Key Laboratory of Cell Proliferation and Regulation Biology, Ministry of Education, College of Life Sciences, Beijing Normal University, Beijing 100875, China; 6 Key Laboratory of Prevention, Diagnosis and Therapy of Upper Gastrointestinal Cancer of Zhejiang Province, Hangzhou 310022, China

**Keywords:** inter-organelle communication, membrane contact sites, optogenetics, proximity labelling

## Abstract

Membrane contact sites (MCSs) mediate crucial physiological processes in eukaryotic cells, including ion signaling, lipid metabolism, and autophagy. Dysregulation of MCSs is closely related to various diseases, such as type 2 diabetes mellitus (T2DM), neurodegenerative diseases, and cancers. Visualization, proteomic mapping and manipulation of MCSs may help the dissection of the physiology and pathology MCSs. Recent technical advances have enabled better understanding of the dynamics and functions of MCSs. Here we present a summary of currently known functions of MCSs, with a focus on optical approaches to visualize and manipulate MCSs, as well as proteomic mapping within MCSs.

## Introduction

**Figure 1.**
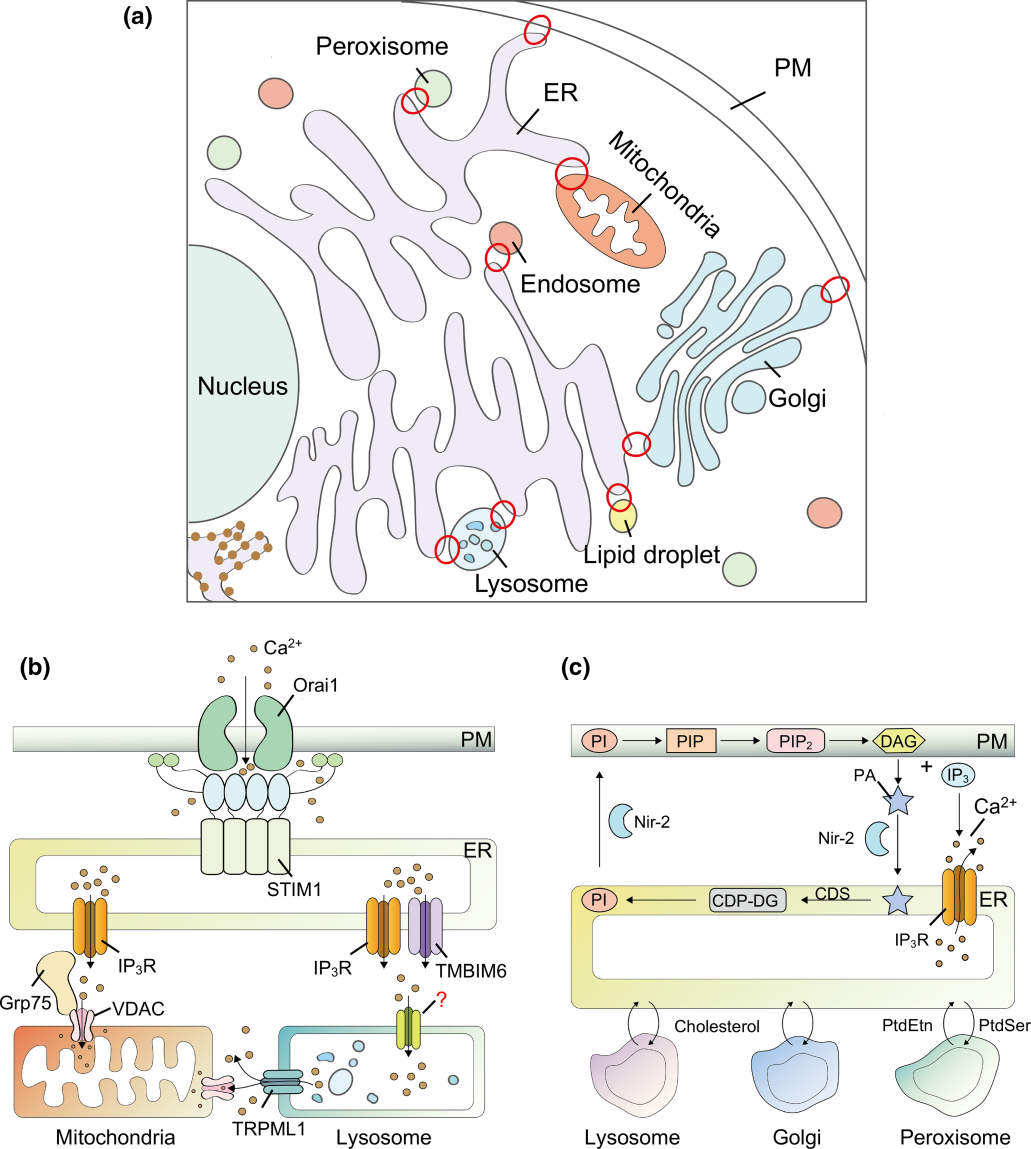
Membrane contact sites (MCSs) in mammalian cells. (**a**) Illustration of MCSs between intracellular organelles (red circles). (**b**) Ca^2+^ mobilization at various MCSs. Schematic representation of SOCE at ER-PM junctions, mitochondrial Ca^2+^ uptake at MAMs, and Ca^2+^ refilling at ER-lysosome interface. (**c**) Lipids cycling at MCSs. Abbreviations: MCSs, membrane contact sites; Ca^2+,^ calcium ions; PM, plasma membrane; ER, endoplasmic reticulum; IP_3_R, inositol triphosphate receptor; GRP75, glucose-regulated protein 75; VDAC, voltage-dependent anion selective channel; TRPML1, transient receptor potential mucolipin channel 1; TMBIM6, transmembrane BAX inhibitor motif containing 6; PI, phosphatidylinositol; PIP, Phosphatidylinositol 4-phosphate; PIP_2_, Phosphatidylinositol 4,5-bisphosphate; DAG, also named DG, diacylglycerol; PA, phosphatidic acid; NIR-2, membrane-associated phosphatidylinositol transfer protein 1; CDS, CDP–DG synthase; CDP-DG, cytidine diphosphate diacylglycerol; IP_3_, inositol 1,4,5-triphosphate; PtdEtn, phosphatidylethanolamine; PtdSer, phosphatidylserine.

Membrane-bound organelles are subcellular compartments within eukaryotic cells, and they enable spatial organization of unique biochemical reactions [1,2]. Membrane contact sites (MCSs) are organellar juxtaposition with distances between tethering membrane structures typically within 30 nm, and can be relatively stable or maintained in a transient state without fusion of membranes (Figure 1a). Communications through MCSs in recent years have been considered as the center of intracellular physiological homeostasis [1]. Specialized proteome or liposome within MCSs enables distinct functions like regulation of fusion between the two organelle membranes, calcium signaling (Figure 1b), lipid biosynthesis and transfer (Figure 1c) [3]. Abnormalities of MCSs may lead to many types of diseases, even cancers (Table 1) [4–6]. Although decades have passed since MCSs were first observed, approaches to accurately visualize contact sites and analyze their compositions are still dismal [7]. Even proteins in specific intracellular regions could be analyzed by traditional proteomic mapping technique and fluorescence microscopy, spatio-temporal analysis of the dynamic proteome of MCSs and visualization of contact sites in living system still remain challenging.

**Table 1:** Summary of MCSs in related diseases

Disease	Related MCSs	Refs.
Type 2 diabetes	ER-Mitochondria	Arruda et al. [22]
Liver disease	ER-Mitochondria	Hernández et al. [23]
Kidney disease	ER-Mitochondria	Inoue et al. [24]
Cardiac arrhythmia	ER-Mitochondria	Boyman et al. [25]
Parkinson's disease	ER-Mitochondria	Area-Gomez et al. [26]
	Mitochondria-Lysosome	Kim et al. [27]
CMT2	Mitochondria-Lysosome	Rossor et al. [28]
LSD	Mitochondria-Lysosome	Platt et al. [29]
Muscle weakness	ER-PM	Andersson et al. [30]
Immunodeficiency	ER-PM	Feske et al. [31]
Stormorken syndrome	ER-PM	Misceo et al. [32]
		Morin et al. [33]
SCID	ER-PM	Carroll et al. [34]
Heart hypertrophy	ER-PM	Fayssoil A. et al. [35]
		Prins et al. [36]
Heart failure	ER-PM	Bartoil et al. [37]
Malignant hyperthermia	ER-PM	MacLennan et al. [38]
Cancer	ER-Mitochondria	Bittremieux et al. [39]
	ER-Lysosome	Saxton et al. [40]
	ER-Endosome	Peretti et al. [41]

Abbreviations: CMT2, Charcot-Marie-Tooth disease type 2; LSD, lysosomal storage disease; SCID, Severe combined immunodeficiency; ER, endoplasmic reticulum; PM, plasma membrane; MCSs, membrane contact sites.

Even though the coordination among organelles is essential for cells to function properly as a unit, previous studies were mostly focused on identifying players and corresponding events within organelles. Over recent decades, complementary techniques such as proximity ligation assay (PLA) [8,9], super-resolution imaging (SR) [10,11], electron microscopy (EM) [12], electron cryo-tomography (Cryo-ET) [13], subcellular fractionation (SF) [14], Bimolecular Fluorescent Complimentary (BiFc) [15], Förster resonance energy transfer (FRET) [16], bioluminescence resonance energy transfer (BRET) [17], as well as biochemical or optogenetic tools have emerged [18–20]. These strategies have been widely utilized to better characterize the molecular compositions of intermembrane communications, visualize, and manipulate the formation of MCSs [21].

This review summarizes recent findings about cellular events, mostly ion transport and lipid biosynthesis occurring in MCSs, as well as these newly adapted approaches for the proteomic-mapping, visualization and manipulation of the dynamic inter-organelle contact sites.

## Calcium and lipid dynamics at inter-organelle membrane contact sites

Calcium ion (Ca^2+^) is a second messenger crucial for many delicate physiological processes pertaining to cell signaling, such as protein folding, hormone secretion, synaptic transmission, programmed cell death, and muscle contraction [42].

The endoplasmic reticulum/sarcoplasmic reticulum (ER/SR) is one of the main intracellular stores of Ca^2+^ and one major role of ER/SR related MCSs is calcium signaling [1,3]. Ca^2+^ is released from the ER/SR into the cytoplasm via inositol triphosphate receptors (IP_3_R) or ryanodine receptors (RyR) [43]. Ca^2+^ signaling at ER-plasma membrane (PM) junctions is among the first identified signaling events in MCSs (Figure 1b). For excitable cells like skeletal or cardiac muscle cells, when they get stimulated, depolarization of PM will activate voltage-operated Ca^2+^ channels, which then further induce Ca^2+^ release from ER via either direct physical coupling or Ca^2+^ induced Ca^2+^ release (CICR), eventually leading to the contraction of muscle cells [44,45]. As to non-excitable cells, activation of PM receptors often leads to ER Ca^2+^ release via phospholipids C and inositol triphosphate (PLC-IP_3_) pathway. After Ca^2+^ store depletion, a process called store-operated Ca^2+^ entry (SOCE) at ER-PM junctions is activated for signaling and ER Ca^2+^ restoration [46,47].

There is also Ca^2+^ transfer from mitochondria-associated membrane (MAM) portion of ER to mitochondria [48,49]. Glucose-regulated protein 75 (Grp75) plays a key role in formation of MAM by linking IP_3_R and voltage-dependent anion selective channel (VDAC) to form the IP_3_R-GRP75-VDAC complex (Figure 1b), thereby regulating the shuttling of Ca^2+^ across outer mitochondrial membranes (OMM) [50]. In addition to the above-mentioned complex, mitofusin 2 (Mfn2) also plays an indispensable role in Ca^2+^ transport at OMM. It is able to anchor to ER and OMM respectively, and promotes the portion of MAM through homotypic interactions [51]. Compared with OMM, inner mitochondrial membranes (IMM) is less permeable to Ca^2+^ [52]. After Gunter et al. [53] revealed the mechanism of sodium-calcium exchange and hydrogen-calcium exchange mediating Ca^2+^ transport at IMM, Kirichok et al. [54] made patch-clamp recording of the IMM and found that Ca^2+^ uptake was mediated by mitochondrial Ca^2+^ uniporter (MCU). Dysregulation of MAM is a major cause of type 2 diabetes mellitus (T2DM). Arruda et al. [22] revealed that increasing MAM ratio in mouse liver cells resulted in the overload of mitochondrial calcium and insulin resistance, thus causing T2DM.

Acidic membrane-bound organelles represented by lysosomes are also indispensable intracellular Ca^2+^ stores [55–57]. Transfer of Ca^2+^ at ER-lysosomes contacts is mediated by Ca^2+^ channels like transient receptor potential mucolipin channels (TRPMLs) and two-pore channel (TPC). TRPMLs, which belong to the transient receptor potential (TRP) channel family, exist in three isoforms and mediate autophagy, in which calcium release through TRPML1 is regulated by lysosomal potassium ion (K^+^) channels [58,59]. The TPC1 and TPC2 isoforms of TPC are anchored to endosome and lysosome, respectively, and modulate assembly of ER-lysosome MCSs and the corresponding Ca^2+^ transport [60,61]. A class of phosphatidylinositol, specifically phosphatidylinositol 3,5-bisphosphate (PI(3,5)P_2_) present in the ER-lysosome contacts, also tones lysosomal Ca^2+^ stores by regulating TRPML and TPC [62]. It has been reported that Ca^2+^ refilling of lysosome is dependent on the Ca^2+^ levels inside the ER, as well as IP_3_Rs on its membrane. The Ca^2+^ exchange is achieved through ER-lysosome MCSs [63]. The high concentration of Ca^2+^ contributed by IP_3_R-mediated calcium efflux from the ER provides trigger conditions for lysosomal Ca^2+^ refilling, and theoretically any calcium channels on lysosomal membrane can mediate Ca^2+^ transport [64]. In addition, calcium release at MCSs can activate voltage-gated K^+^ channel to promote refilling [59]. However, the identity of specific channel on lysosome for the Ca^2+^ refilling still remains elusive (Figure 1b) [65]. In addition, TRPML1 may also mediate Ca^2+^ transport from lysosomes to mitochondria via mitochondria-lysosome contact sites (Figure 1b) [66].

The other prominent role of MCSs is for lipid biosynthesis and exchange [3]. Lipid, the primary component of cellular membrane, is critical to many cellular operations that require the condition of membrane lipid allocation being properly maintained [67]. Lipid is usually considered as the trigger or terminus of biochemical process in the cells, for instance, phosphatidylinositol (PI)-phosphatidic acid (PA) cycling between ER and PM with the generation of significant second messengers, inositol 1,4,5-triphosphate (IP_3_) and diacylglycerol (DAG) (Figure 1c) [68].

Phosphatidylserine (PS) transport between ER-mitochondria has been shown to be associated with MAM, but the mechanism of phospholipid and sterol transport between other organelles is still poorly understood [69]. Recent studies have revealed the mechanism of low-density lipoprotein (LDL) transport from lysosome first to PM and then to ER via ER-PM contact sites [70,71]. After screening with CRISPR-Cas9 technology, a strong correlation with sterol regulatory element-binding protein 2 (SREBP-2) process was identified for *Phosphatidylserine synthase 1* (*PTDSS1*), a gene encoding PS synthase. PTDSS1 deficiency would cause down-regulation of PS level [71]. Trinh et al. [71] found that LDL-derived cholesterol transport from PM to ER at ER-PM contact sites was impaired in PTDSS1-deficient cells, resulting in its retention at PM, but LDL uptake and degradation remained normal. Furthermore, studies on the GRAMD1/Aster protein family have shown that these ER-anchored proteins bind cholesterol and negatively charged lipids in PM (e.g. PS) via Aster and GRAM domains, respectively, and further induce ER-PM MCSs formation for cholesterol transporting from PM to ER. Thus, the lack of PS prevents the formation of MCSs in PTDSS1-deficient cells, which leads to the accumulation of cholesterol in PM [70–72].

Triglyceride (TG) accumulation in hepatocytes is a major pathogenic cause of non-alcoholic fatty liver disease [73]. The presence of diacylglycerol acyltransferase (DGAT) [74], PS synthase [75], and cholesterol acyltransferase [76] in MAM is well documented for their important roles in intracellular lipid exchange. As described above, Grp75 possesses a pivotal role in the formation of MAM. To further elucidate roles of Grp75 in lipid metabolism, Bassot et al. altered the expression of Grp75 and Mfn2 in cells. Down-regulation of these two proteins led to decreases in amount of MAM portion and β-oxidation capacity of fatty acid in the mitochondria, which in turn caused the accumulation of TG inside hepatocytes [73].

Dysregulation of Ca^2+^ signaling and lipid metabolism is inextricably linked to cancer. Store-operated calcium entry (SOCE) has an indispensable role in progression of many types of cancer [5]. Its up-regulation inhibits the YAP/TAZ pathway to suppress the growth of glioblastoma [77]. Meanwhile, the down-regulation of SOCE reduces metastasis of breast and liver cancer [78,79]. Ca^2+^ transport to mitochondria activates the tricarboxylic acid cycle and affects the Warburg effect in cancer cells [80]. Therefore, altered Ca^2+^ signaling at MAM will affect apoptosis [39]. Lipids are energy reserves of cells and are transported via multiple lipid transport proteins (LTP), most of which require the assembly of MCSs for their function [81,82]. It has been reported that LTP dysfunction will induce cancer development [41]. In ER-Golgi MCSs, Nir2 regulates ceramide trafficking by interacting with ceramide transfer protein (CERT, a ceramide channel) and oxysterol-binding protein (OSBP) [83]. Furthermore, the regulation of lipid transport via Nir2 is important for the activation of phosphatidylinositol 3-kinase (PI3K) pathway, thereby affecting breast cancer progression [84].

## Proteomic proximity-labeling for mapping MCSs components

Although decades have passed since MCSs were observed with traditional EM, the acquisition of fine structures and dynamics of MCSs still remains challenging. And even proteins in specific intracellular regions can be analyzed by traditional biochemical techniques, it is extremely difficult to perform proteomic mapping of these juxta-membrane regions. Traditional proteomic mapping approaches, such as co-immunoprecipitation, involve disruptive manipulations like cell lysis and purification of subcellular fragments. Protein machineries within these juxta-membrane regions could easily get lost during such harvest processes, hindering proteomic mapping of MCSs.

**Figure 2.**
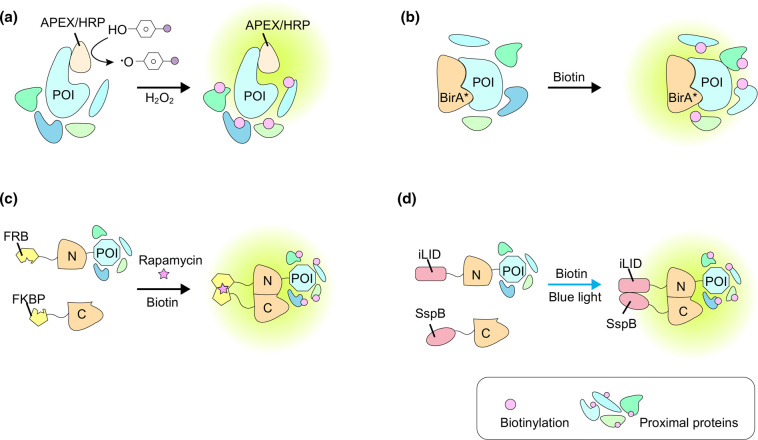
Proteomic proximity labeling approaches. Labeling area indicated by circular glows in green. (**a**) Peroxide-based methods, in the presence of hydrogen peroxide, APEX/HRP will convert biotin phenol to biotin phenol radicals, which then diffuse out and label nearby proteins. (**b**) Biotin ligase-based methods for proximity labeling. (**c**) Mode of chemical induced split-TurboID. The biotin ligase is split into two inactive fragments. To restore its function, the two parts need to be joined-back together by rapamycin-induced dimerization. (**d**) Cartoon representation of light–controlled re-assembling of split TurboID, termed OptoID. The iLID/SspB light control system is installed onto split-TurboID to bring two parts in close proximity upon blue light irradiation. Abbreviations: APEX, ascorbate peroxidase; HRP, horseradish peroxidase; BirA*, BirA-R118G; BioID, proximity-dependent biotin identification; POI, protein of interest; FKBP, FK506 binding protein; FRB, FKBP12-rapamycin binding; OptoID, photo-sensitive split-TurboID; N, N-terminal of TurboID; C, C-terminal of TurboID.

Nowadays, the advent of proximity labeling technology coupled with mass spectrometry (MS) makes it feasible to compile proteomic mapping in living cells. Proximity labeling involves enzymatic catalytic reactions to covalently label neighboring proteins with biotin within its radius (∼10–20 nm) [85]. Horseradish peroxidase (HRP), ascorbate peroxidase (APEX), and proximity dependent biotin identification (BioID) are major enzymes utilized for proximity-labeling (Figure 2a,b). Unlike traditional approaches, they can capture proteomes in natural cellular state. Hence, characterization of the proteome within MCSs will not be affected by isolation and purification processes of proteins [86,87].

In the presence of hydrogen peroxide (H_2_O_2_), HRP can catalyze the generation of biotin phenol or biotin-aryl azide radicals for proximity labeling [88–90]. HRP has disulfide bonds, thus needing oxidizing environment like the luminal of oxidizing organelles such as ER, Golgi or extracellular area to maintain its proper structure and exert proximity labeling activity [91,92]. On the contrary, in cytoplasm, HRP is inactive due to loss of proper structure induced by the reducing environment, which limits its application in the analysis of MCSs and promotes the development of APEX [89]. Although HRP cannot process proximity labeling of MCSs, it can be combined with EM to detect the structure of MCSs. HRP catalyzes the polymerization of 3,3′-diaminobenzidine (DAB), which can be used as a marker for EM imaging after immobilization with osmium tetroxide to identify the membrane of organelles [89,93]. Schikorski et al. successfully transfected HRP cDNA into neuronal cells as a reporter gene and analyzed their ultrastructure under EM. They added nucleotide sequence encoding human growth hormone secretory signal and ER retention signal to HRP cDNA to ensure HRP glycosylation and localization in the ER. This engineered, ER-localized, HRP could thus provide an unambiguous label of ER, and it could even be used to detect the integrity of the ER ultrastructure in neurons [93].

Unlike HRP, APEX can preserve its activity in reducing environment due to the lack of disulfide bond and calcium binding domains [94]. In the presence of H_2_O_2_, APEX catalyzes the generation of biotin phenol radicals that covalently tags neighboring proteins, which are short-lived (<1 ms), though the experiments may take tens of seconds for complete tagging (Figure 2a) [86]. This fast-labeling approach enables mapping proteomic changes in cellular reactions. Among the application scenarios of APEX, subcellular region analysis is most prominent [95]. The catalytic activity with low expression of APEX system is too low to allow sufficient amount of biotin tagging, while too much overexpression of APEX will generate non-specific labeling. To overcome these drawbacks, Lam et al. utilized fluorescence activated cell sorting (FACS) screening, identified an A134P mutant of APEX and named it APEX2. APEX2 showed higher catalytic activity at low concentrations and achieved better proteome enrichment than its prototype [94]. By tagging APEX2 onto STIM1, Jing et al. successfully obtained the proteomics at ER-PM junctions both before and after SOCE. This is the first report of proteomic mapping of MCSs using proximity labeling [18]. Meanwhile, APEX has also been applied *in vivo* to label subcellular compartments and other proteins as a way to reveal a complex network of signaling interactions. Representative ones include: lipid droplets [96], endosomes [97], ER-mitochondria MCSs [98] and G protein-coupled receptors [99], voltage-gated calcium channels [100], and cell growth factors [101]. However, the need for H_2_O_2_ is one major limitation of these peroxidases for proximity labeling in live cells because of its toxicity and signal cross-talking.

An alternative approach is to use less harmful tools that are based on biotin ligase. BioID-based proximity labeling utilizes mutants of a biotin protein ligase called BirA [102]. In 2005, Cronan et al. [102] discovered that BirA-R118G allowed the release of its catalytic product, biotinyl-5′-AMP, to its surroundings, enabling biotinylation of its neighboring proteins. Roux et al. renamed BirA-R118G as BioID and successfully used it to obtain proteomics in animal cells [87,103]. Even though the BioID approach is nontoxic to cells, its tagging efficiency is relatively low. It takes almost 24 h to ensure adequate tagging of the surrounding proteins. This long-period of labeling reduces the spatiotemporal resolution of the obtained proteomics, making it undesirable for dynamic proteomic mapping within MCSs [87]. Later, BirA mutants with faster kinetics, including miniTurbo, TurboID [104] and BASU [105], were developed. They allow proximity labeling in tens of minutes or as fast as one minute.

Even though the aforementioned methods are suitable for identifying weak or transient interactions between proteins within MCSs, they share some intrinsic drawbacks that restrict their applications. For example, they lack the specificity for identifying protein complexes, especially for those spatiotemporally dynamic protein–protein interactions (PPI). Moreover, their temporal resolution may be hindered by the basal biotinylation activity due to the existence of endogenous biotin, and their spatial resolution is restricted by the subcellular localization of the protein it fused on [106–108].

To overcome these issues, series of split-ID methods have been developed recently. All of these tools share the same design strategy [106–108]. First, with a combination of protein engineering and other approaches, find a ‘cut' in the proximal labeling enzyme, so that either part of its ‘half' is not functional by itself. Only when these two parts are not only co-expressed, but also in close proximity and correct orientation, they can then join back together to make a functional labeling enzyme. Second, fuse the corresponding coding sequence (CDS) of these two ‘half' enzymes onto CDS of two proteins that are supposed to interact or be localized on two separate sides of one given type of MCS. Third, co-express both components of this tool set into cells. And cells co-expressing these two halves would then enable specific proximal labeling of target protein complexes or MCSs after two parts assembled, and the labeling process stops when they get separated, making the labeling specific for protein complexes or MCSs on which the tool is tagged [106–108]. This type of tools thus allows verification of protein interactions and analysis of the proteins communicating with the complex.

Split-BioID is the first developed Split-ID tool. To overcome the deficiencies in protein affinity purification (AP) and mass spectrometry (MS) identification in probing protein interactions crucial for miRNA-mediated gene silencing, Schopp et al. [106] generated split-BioID based on BioID. They used protein fragment complementation assay (PCA) to determine the optimal E256/G257 split site and to make the almost non-functional N-BirA* and C-BirA* halves [106]. By linking the two halves to 14-3-3ɛ and Cdc25C, respectively, the team then used the resulting split-BioID system accurately identified additional interacting factor, such as the interaction of LMO7 with Cdc25C/14-3-3ɛ dimer. They also revealed that the degree of biotinylation of LMO7 was dependent on Cdc25C/14-3-3ɛ dimerization leading to the restoration of the biotin ligase activity of split-BioID [106]. They next established Argonaute (Ago) and miRNA-induced silencing complex (miRISC) function differently in cells and identified Ago downstream effector CCR4/NOT complex with split-BioID [106]. Furthermore, Rhee's group was the first to identify G78/G79 as cleavage site in wild-type BirA based on Debye–Waller factors (B factors), developing a split-BioID system named Contact-ID [108]. Compared with Split-BioID, Contact-ID has better biotinylation catalytic activity and does not exert activity at all when the two fragments are present alone [108]. However, since the above methods are all modified based on the BioID, the slow kinetics (at least 16 h is required to detect the signal) has become an inevitable defect [107].

To enable specific proteomic mapping of MCSs with a fast speed, Alice Y. Ting's group developed a novel proximity labeling tool called split-TurboID, which decomposed TurboID, a faster version of BioID, into two catalytically inactive fragments (Figure 2c, Table 2) [107,109]. When co-expressed in cells, these two components of split-TurboID were localized on different subcellular membrane. Only when MCSs were formed, the two spliced parts could then join back together to restore labeling activity in MCSs, and addition of rapamycin further enhanced the interactions between the two parts, thus enabling specific proteomic mapping of MSCs [107]. Takano et al. introduced the split-TurboID system to analyze proteins at the contact sites of astrocytes and neuronal membranes both *in cellulo* and *in vivo*. They determined that astrocytes regulated the formation of inhibitory synapse through neuronal cell adhesion molecule (NRCAM) that could bind to gephyrin at inhibitory synapses, indicating a key role in γ-aminobutyric acid (GABA)-induced neural inhibition [110]. Unlike the cytotoxic APEX labeling method [110], split-TurboID is better suited for resolving activity dependent proteomics *ex vivo* or *in vivo*.

**Table 2.** Characterization of split biotin-based proximity labeling approaches

Approach	Biotin ligase	Split site	Inducer	Refs.
split-BioID	BioID	E256/G257	Rapamycin	Schopp et al. [106]
split-TurboID	TurboID	L73/G74	Rapamycin	Cho et al. [107]
SUMO-ID	TurboID	T194/G195	SUMO-SIM interaction	Barroso et al. [113]
Opto-ID	TurboID	G99/E100	Blue light	Chen et al. [112]

Abbreviations: BioID, proximity-dependent biotin identification; OptoID, photo-sensitive split TurboID; SUMO, Small Ubiquitin-like Modifiers; SIMs, SUMO interacting motifs.

One drawback of split-TurboID is that the efficient restoration of its catalytic activity needs the presence of rapamycin. This chemical may induce undesired cellular responses through activation of the mTOR signaling pathway [111]. To further improve the efficiency and specificity of labeling in MCSs, optogenetic version of split-TurboID has been developed. Chen et al. developed an optogenetic tool called OptoID (photo-sensitive split TurboID). By inserting a variant of the photo-induced dimer system at the G99/E100 site of the TurboID (Figure 2d, Table 2), the resulting sliced-parts of OptoID could be joined back together by light. Thus, under the irradiation of blue light, OptoID exhibited high biotinylation activity, addressing the spatial limitations of chemically inducible methods and reducing the background of TurboID in response to endogenous biotin [112]. The introduction of light-sensitivity enables reversible controlling the activity of OptoID, demonstrating its potential for highly accurate proteomic mapping of MCSs.

## Approaches to visualize MCSs in fixed samples and living cells

EM is considered as the golden standard for MCSs visualization, as EM utilizes HRP or colloidal gold to localize membrane proteins, enabling the detection of MCSs at nanoscale [21]. However, to observe MCSs via EM, vacuum conditions, along with dehydration staining to enhance the resolution, are needed. Furthermore, sample damage caused by collisions and heating of electron-beam are inevitable. Thus sub-cellular structures revealed with EM could either be distorted, or even contain some artifacts [13]. The emergence of Cryo-ET addresses these deficiencies, as this technique freezes the cell in non-crystalline ice, causing almost no harm to the structure of cellular proteins as well as subcellular compartments. Thus Cryo-ET enables high-resolution detection of three-dimensional structure of MCSs under the closest natural conditions of the cell [13]. FIB-SEM, an instrument that combines the usage of focused ion beam (FIB) and scanning electron microscope (SEM) [114]. FIB-SEM is able to grind individual slices to a thickness of ∼10 nm and maintain co-localization of cutting and imaging in serial sectioning process, avoiding section defects and artifacts, further displaying overall structures and subcellular details of tissues [115,116]. Despite all these improvements and their nanoscale resolution, the application of these techniques is restricted by the need for special equipment and laborious sample preparation.

**Figure 3.**
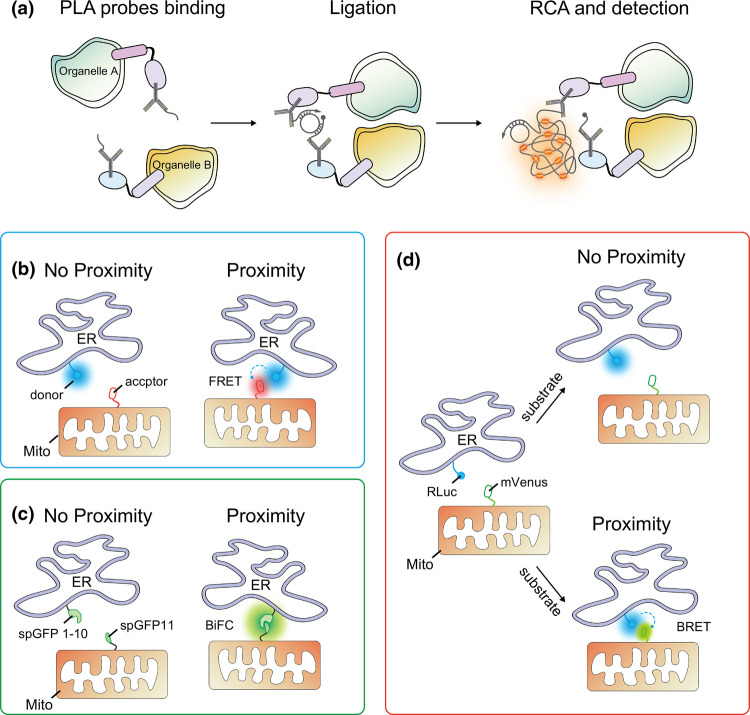
Fluorescence/Bioluminescence-based approaches to label MCSs. (**a**) Cartoon illustration of the proximity ligation assay (PLA). Two single-stranded oligonucleotides conjugated antibodies recognize specific proteins located on two different organelles, respectively. When two organelles are in close proximity, connector oligos join the PLA probes and become ligated. The signal of circular DNA molecule is amplified by rolling-circle amplification (RCA). (**b**) Förster resonance energy transfer (FRET). Since the accessible proximity between two different organelles, the Förster resonance energy is transferred from the excited donor to the acceptor. (**c**) Principle of bimolecular fluorescence complementation (BiFC) analysis in MCSs detection. Accessible proximity between two opposing membranes of different organelles makes GFP1-10 and GFP11 reassembly and fluorescence restoration. (**d**) Scheme illustrating bioluminescence resonance energy transfer (BRET). In response to substrate treatment, significant BRET between blue-emitting luciferase (Rluc) and acceptor fluorophore (mVenus) indicates proximity of the two different organelles. Abbreviations: RCA, rolling circle amplification; ER, endoplasmic reticulum; Mito, mitochondria; Rluc, Renilla Luciferase 8.

Alternatively, proximity-driven fluorescent approaches, such as PLA, FRET, BRET and BiFC, have been applied to identify the potential MCSs using regular fluorescence microscopy with a lower resolution. PLA was frequently used for visualizing and detecting endogenous interacting pairs of PPI in fixed cells or tissues [117]. Nowadays, PLA has also been explored to discover the key elements involved in inter-organelle contact sites (Figure 3a). It was found that StAR-related lipid transfer domain-3 (STARD3) and STARD3 N-terminal like protein (STARD3NL), were involved in the MCS assembly between late endosomes (LE) and ER [118]. In addition, Gomez-Suaga et al. [8] also examined the assembly of ER-mitochondria tethering via PLA, and found that overexpression of vesicle-associated membrane protein-associated protein B (VAPB) and protein tyrosine phosphatase interacting protein 51 (PTPIP51) remarkably enhanced ER-mitochondria MCSs formation to take part in Ca^2+^ exchange and autophagy. Overall, the PLA technique is highly sensitive, as it utilizes antigen-antibody reactions to greatly amplify the signal. Therefore, it is suitable for the detection of sparsely distributed MCSs.

The EM approaches and PLA could only be applied to fixed cells or tissues. However, the specific sample preparation requirements of above techniques make them inapplicable for monitoring the dynamics of MCSs in living cells. FRET and BiFC techniques may serve this purpose. In general, by tagging fluorescent proteins onto known proteins within MCSs, the resulting fluorescently labeled MCSs could then be visualized under the microscope [119].

The spatial resolution of all these fluorescent or FRET-based tools is defined by the imaging equipment used. Compared with a lateral resolution of roughly 200 nm, the longitudinal resolution of a typical confocal microscope is usually not as good, ∼500 nm [120]. This poor resolution over *Z*-axis can be improved by total internal reflection fluorescence microscopy (TIRFM), which has a longitudinal resolution smaller than 200 nm [21]. TIRFM uses the total reflection of light at the interface between water and glass to produce an excitation light called evaporation wave to selectively illuminate the surface of the PM-related MCSs [121,122]. The effective penetration depth of the probe wave is maintained within 100 ∼ 200 nm, avoiding the visualization of fluorescent proteins or structures deep within the cells, thus improving the accuracy [123]. Nevertheless, the resolution of confocal or TIRFM is one order of magnitudes larger than the size of MCS, severely hindering the dissection of MCS dynamics.

In recent years, a series of super-resolution (SR) imaging techniques have emerged that break the limits of optical diffraction. By taking advantage of the nonlinear properties of light and computational reconstructions, super-resolution (SR) microscopy can increase the resolution to tens of nanometers [124]. Current SR techniques include stimulated emission depletion microscopy (STED) [125], single molecule localization microscopy (SMLM) [126], structured illumination microscopy (SIM) [9], photoactivation localization microscopy (PALM) [10], and stochastic optical reconstruction microscopy (STORM) [127]. SR imaging, especially SIM with relatively fast acquisition speed, has become a powerful tool to visualize and study MCSs in living cells (Table 3) [120], especially with the help of fluorescent-labeling tools that specifically localize to certain types of MCSs. For example, Liou's group developed a fluorescent tag for ER-PM MCSs named membrane attached peripheral ER (MAPPER) [128]. MAPPER could be considered as an engineered truncated STIM1 mutant, with the signal peptide (SP) and the transmembrane (TM) domain of STIM1 to enable its ER localization, the polybasic (PB) motif from the small G protein Rit to allow the binding with the acidic PI on PM by electrostatic interactions, and a proper-sized linker between these two STIM1 fragments. Thus, when expressed at low levels, MAPPER would preferably locate to ER-PM MCSs. However, high expression of these types of tools may artificially expand the size of MCSs, and the resolution of these SR imaging techniques is not powerful enough to resolve MCSs structures, further improvements are needed.

**Table 3.** Exemplary techniques and the resolution for MCSs visualization.

Technique	Lateral resolution	Axial resolution	Refs.
Cryo-ET	∼1–4 nm	∼1–4 nm	Gan et al. [132]
FIB-SEM	<10 nm	<10 nm	Xu et al. [133]
Confocal	∼200 nm	∼500 nm	Schulz et al. [120]
TIRFM	∼200 nm	∼100 nm	Jin et al. [134]
			Mattheyses et al. [123]
STED	30–80 nm	∼100 nm	Hell et al. [125]
			Wildanger et al. [135]
SMLM	20–30 nm	50–60 nm	Huang et al. [138]
SIM	100 nm	250 nm	Sengupta et al. [10]
PALM	Up to 20 nm	50 nm	Hirabayashi et al. [9]
STORM	Up to 20 nm	50 nm	Rust et al. [127]

Abbreviations: Cryo-ET, cryo-tomography; FIB-SEM, focused ion beam scanning electron microscope; TIRFM, total internal reflection fluorescence microscopy; STED, stimulated emission depletion microscopy; SMLM, single molecule localization microscopy; SIM, structured illumination microscopy; PALM, photoactivation localization microscopy; STORM, stochastic optical reconstruction microscopy.

Besides these MCS-localized, single-component fluorescent markers, BiFC is another type of fluorescent-based tools to label MCSs. Split Venus [129] or spGFP1–10/spGFP11 [130] could be fused to each of two proteins located on the opposing membranes of inter-organelles. If the two organelles are close enough, they may bring the two split fluorescent fragments into close proximity (10–30 nm) to restore the fluorescence (Figure 3c) [131]. BiFC offers a straightforward readout to identify unknown binding partners within MCSs. With this strategy, the dynamic contact sites between ER and mitochondria have been demonstrated under various physiological conditions in mammalian cells [15]. Unlike MAPPER-type tools that are also fluorescent outside MCS, BiFC signal only exists in MCSs, thus BiFC is more specific for MCS probing. However, unlike the PLA approach that could generate large signals, BiFC signal is weaker. Thus, even though BiFC is compatible with live-cell imaging, it suffers from contamination from auto-fluorescence.

The advent of FRET has enabled nanometer-scale resolution of MCSs dynamics, as FRET is a phenomenon that occurs only between two fluorophores (a donor and an acceptor) when the distance between them is smaller than 10 nm. Briefly, when the donor fluorophore gets excited by illumination, without generating its own fluorescence, it instead transfers energy to its acceptor chromophores to make the latter fluorescent, and return itself to ground state (Figure 3b) [137,138]. FERT has become a powerful tool for studying MCSs. By combining FKBP-FRB and FRET, rapamycin-induced activation of FRET signaling can be achieved for analysis of ER-mitochondrial MCSs and Ca^2+^ level alterations in myocardium [137]. Perhaps the biggest advantage of FRET-based tools is that it is more quantitative. FRET efficiency is inversely dependent on the distances between the donor and acceptor. After careful calibration, the calculated FRET efficiency indexes could be a direct indicator of the distances between MCS membranes, in dependent of expression levels of the tool and instruments used [139,140].

However, photobleaching, and direct excitation of the acceptor fluorophore limit the application of fluorescent-based approaches. Bioluminescence -based biosensor, BRET, could avoid such problems. Even though BRET also use FRET to transfer energy to the acceptor, the energy for donor excitation comes from bioluminescence. A luciferase enzyme in proximity of acceptor would catalyze a bioluminescent oxidation in response to substrate treatment, providing the energy required for the activation of donor (Figure 3d). Recently, mitochondria–ER length indicator nanosensor (MERLIN) has been developed to reversibility analyze the dynamic contact between ER and mitochondria [141]. Hence, BRET offers broad applications in radiation-free energy transfer and avoids the consequences of fluorescence excitation to identify new tethers involving in the MCSs.

Overall, the above-mentioned emerging approaches are powerful tools to visualize and label the MCSs in fixed samples and living cells, each with advantages and disadvantages of their own. Besides these visualization approaches, powerful and innovative tools that can reversibly manipulate MCSs assembling and signal transduction, are also crucial for researches on MCSs.

## Approaches to manipulate MCSs assembling and signal dynamics

**Figure 4.**
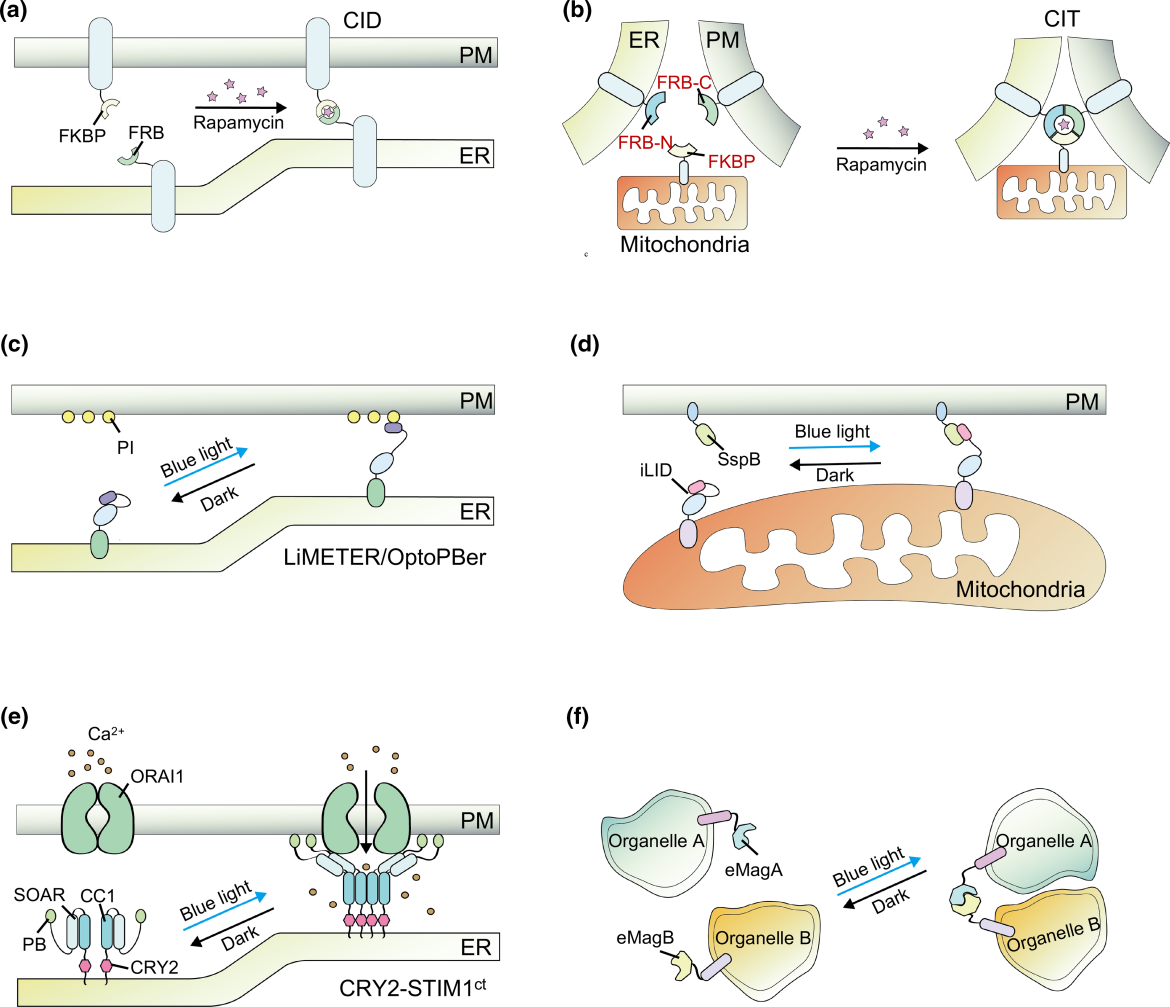
Labeling and manipulations of MCSs by chemicals or light. (**a** and **b**) Chemical-induced MCSs assembling through CID and CIT system. CID- and CIT-mediated dimerization of fragments targeting to ER and PM (**a**), or trimerization of those targeting to ER, PM, and mitochondria, respectively, via rapamycin for MCSs (**b**). (**c**–**f**) Light-induced MCSs assembling. (**c**), Schematics of the LiMETER/OptoPBer design. (**d**), iLID employs light switch LOV2 with SsrA/SspB peptides to manipulate ER and mitochondria interactions. (**e**), Manipulation of ER-PM MCS and Ca^2+^ signaling with CRY2-STIM1ct utilizing CRY2 and cytoplasmic domain of STIM1. Induction of CRY2 dimerization under blue light irradiation activates STIM1ct, leading to Ca^2+^ influx through ORAI1. (**f**), eMags-mediated assembling of MCSs. The eMagA and eMagB counterparts are anchored to membranes of two different organelles, respectively. Blue light stimulation induces eMags dimerization to trigger junction formation. Abbreviations: CID, chemically inducible dimerization; CIT, chemically inducible trimerization; ER, endoplasmic reticulum; PM, plasma membrane; LiMETER, light-inducible membrane-tethered peripheral; FKBP, FK506 binding protein; FRB, FKBP12-rapamycin binding; PI, Phosphatidylinositol; iLID, improved light-induced dimer.

Among the currently available molecular tools for remote manipulation of MCSs by chemicals or light, chemically inducible dimerization (CID, Figure 4a) was firstly developed to realize the manipulation of MCSs. One such example is the rapamycin-mediated heterodimerization of FRB fragments of mTOR protein and FK506 binding protein (FKBP) [142–144]. Thus, when FKBP and FRB are targeted to PM- and ER-localized protein, respectively, the introduction of rapamycin can specifically trigger the formation of stable MCSs between ER and PM, and the distance of the gap can be adjusted by introducing linkers of different lengths (Figure 4a) [145].

Based on the CID method, Wu et al. split FKBP and FRB into two parts respectively and realized the development of a chemically inducible trimerization (CIT, Figure 4b) system in the presence of rapamycin. Applying this system, they achieved the formation of the ER-PM-mitochondrial triplex contact site, providing a new idea for the formation of chemically induced triplex MCSs [146]. However, as chemical-inducible methods are irreversible, they may induce adhesion between the affected membranes rather than the formation of a classical MCSs structure, thus preventing precise spatial and temporal regulation of the formation of MSCs [21].

To expand the control range and efficiency of MCSs, optogenetic tools, which could offer reversibility and high spatial and temporal accuracy, have been introduced to label and manipulate MCSs assembling. In 2015, Jing et al. [18] developed an optogenetic tool that reversibly labeled the ER-PM junctions, called light-inducible membrane-tethered peripheral ER (LiMETER, Figure 4c). LiMETER contains an ER-resident domain, light-sensitive module, and a PM-binding fragment. The ER domain of this tool consists of a single TM domain from STIM1 and a signal peptide, with GFP inserted between them as a marker. Its cytoplasmic domain is composed of a light switch named light oxygen voltage-sensing (LOV), and a freely adjustable linker with a PM-targeting PB domain attached to the LOV2 domain [18]. The PB domain contains positively charged residues can bind with PI on PM by electrostatic interaction [147]. Under dark conditions, the Jα helix of LOV2 hides the binding site of PB domains and inhibits its activity. With the stimulation of blue light, Jα undergoes unwinding and undocking from the core domain of LOV2 releasing PB to bind to PI within PM [18]. This was the first optogenetic tool that enabled reversible labeling of ER-PM junctions within seconds, allowing the dynamic visualization of changes in MCSs. Meanwhile, so as to induce the formation of MCSs using optogenetic methods, similar to LiMETER, He et al. [20] constructed an ER-tethered protein based on STIM1-PB and PB, termed OptoPBer, to label ER-PM MCSs under the stimulation of blue light (Figure 4c). An improved light-induced dimer (iLID) also successfully allows reversible manipulation of ER-mitochondrial MCSs formation (Figure 4d). The iLID system enables the assembling of MCSs in a fixed region under blue light irradiation, and prevents the formation of MAM swaths. Moreover, it can still show high light-sensitive activity after multiple light and dark alternating stimulations, exhibiting nice spatial and temporal accuracy [19].

Besides tagging and manipulation of MCSs, multiple tools have been recently developed to gain control of signaling within MCSs, especially Ca^2+^ signals. As the most characterized signaling machinery within MCSs, CRAC channel is the target of optogenetic manipulations. Classical CRAC channels are composed of STIM1 and ORAI1. The calcium-sensing protein STIM1 at ER interacts with the Ca^2+^ channel ORAI1 on PM to mediate SOCE, a major Ca^2+^ influx route in most animal cells [148]. After sensing Ca^2+^ depletion in ER, STIM1 molecules will migrate to the ER-PM junction, where their C-terminal domain unfold and expose the highly conserved STIM-ORAI activating region (SOAR), which then bind with and activate ORAI1 channel unit located on the PM, resulting in Ca^2+^ influxes [149].

A number of optogenetic manipulations have been applied to install light-switching ability into STIM1, the switch of CRAC channels, such as BACCS variants, LOVS1K, OptoCRAC, LOVSoc, OptoSTIM1, monSTIM1, and eOS1 [150,151]. Ma et al. [152] designed a series of tools for probing key molecular factors of STIM1 metamorphosis. By ER-tethered CRY2-STIM1ct (the cytoplasmic domain of STIM1), they achieved light-induced mimic STIM1-puncta at MCSs of ER-PM to trigger Ca^2+^ influx (Figure 4e) [152]. He et al. [153] manipulated CRAC channel by combining LOV2 domain with STIM1 fragment to develop an optogenetic system, LOVSoc, which could be applied to immune cells to manipulate their function. Due to the poor penetrating ability of blue light, there are limitations for the application of blue light systems in deep tissues. Although implantable devices such as photoprobes have been developed and optimized, they still suffer from tissue damage and low coupling efficiency [154]. The introduction of upconversion nanoparticles (UCNP) that can be excited by near-infrared (NIR) and emits light in the wavelength range from UV to NIR wavelengths, is a considerable solution to above problems. The deeper penetration of NIR eliminates the need for optical fiber implantation, allowing direct optogenetic manipulation of deep tissue [155]. With the introduction of lanthanide-doped UCNP, the LOVSoc is remotely activated by NIR, leading to Ca^2+^ influx and resulting immunoinflammatory responses to specifically destruct tumor cells. LOVSoc has prospect of being extended for usage in other non-excitable cells *in vivo*as a mean of regulating Ca^2+^-dependent physiological processes [153]. Furthermore, Kim et al. [156] developed ultra-light-sensitive optogenetic approach, monSTIM1, by using a variant with a modified C-terminal 9-mer peptide (CRY2^Clust^) to successfully induce Ca^2+^ signals via non-invasive light delivery in neurons and astrocytes of mouse brain.

In addition, Fan et al. developed a light-induced tethering (LIT) system using Magnets, another fascinating optogenetic dimerization tool derived from the photoreceptor Vivid of Neurospora crassa. They generated positively- and negatively charged fragments of pMag and nMag by introducing complementary charges in Vivid. The dimerization of above fragments is dependent on simultaneous activation via blue light, ensuring low dark activation. Magnets have low dimerization efficiency and poor expression around human body temperature (37°C) [157,158]. Benedetti et al. thus developed enhanced Magnets called eMags that overcame these shortcomings. Light-activated eMags successfully induced ER-mitochondria and mitochondria-lysosome tethering in HeLa cells with faster kinetics than Magnets. They further developed eMags-based tethers that could regulate the MCSs of the ER-transGolgi network (TGN) and precisely control PIP-cholesterol exchange in a light dependent manner (Figure 4f) [158].

## Conclusions and future prospects

The past decades have witnessed tremendous advances in the understanding of MCSs by microscopic techniques, optogenetic and proteomic techniques. Proximity labeling approaches have helped identification of new players within MCSs, new fluorescent and optogenetic labeling tools of MSCs together with super-resolution imaging have enabled the visualization of the dynamics of MCSs within live cells. Recently developed chemical-genetic and optogenetic tools also enable precise control of MCSs. With the continuous development of switchable optogenetic tools, dynamic control of MCSs with high spatiotemporal precision may be more convenient, and may serve as potential tools for precision medicine. For instance, He et al. [159] applied light-operated Ca^2+^ channel (LOCa) to ER-PM junctions of neurons in drosophila, and successfully alleviated the syndromes of neurodegeneration in a drosophila model of amyloidosis. In exploring the pathogenesis of Parkinson's, Kim et al. [27] found that in dopaminergic neurons, prolonged presence of mitochondria-lysosomal (M-L) contact sites trigger dysregulation of mitochondria distribution and depletion of ATP at axons. Meanwhile, MAM plays a role in regulating mitochondrial calcium homeostasis and mitochondrial activity in Parkinson's disease [160], as well as associations with the pathogenesis of Alzheimer's disease [161].

However, the clinical application of optogenetics remains challenging. Firstly, the application of optogenetic therapy requires gene transfection, which is technically and ethically difficult [162]. Secondly, approaches that could enable penetration of deep tissues are needed, like optogenetic tools that could be excited with infrared or far-red light or up-converting nanoparticles, *etc.* [162]. Thirdly, the treatment of some diseases may require lifelong expression of optogenetic proteins, while the continuous expression of optogenetic genes *in vivo* still remain a challenge. Finally, the introduction of foreign molecules will most likely cause an immune response in the body, resulting in a long-term immune memory that may prevent operation of transfected optogenetic tools [163]. Despite all these aforementioned challenges, optogenetic engineering offers accurate spatiotemporal regulation of cellular functions, and may serve as potential tools for precision medicine, making the use of a beam of light to treat disease not so far-fetched.

## Abbreviations

Ago: Argonaute
AP: affinity purification
APEX: ascorbate peroxidase
B factors: BirA based on Debye–Waller factors
BiFc: Bimolecular Fluorescent Complimentary
BioID: proximity-dependent biotin identification
BRET: bioluminescence resonance energy transfer
Ca^2+^: calcium ion
Ca^2+^: calcium ions
CDS: coding sequence
CERT: ceramide transfer protein
CICR: Ca^2+^ induced Ca^2+^ release
CID: chemically inducible dimerization
CIT: chemically inducible trimerization
Cryo-ET: cryo-tomography
DAB: 3,3′-diaminobenzidine
DAG: diacylglycerol
DGAT: diacylglycerol acyltransferase
EM: Electron microscopy
ER/SR: endoplasmic reticulum/sarcoplasmic reticulum
FACS: fluorescence activated cell sorting
FIB: focused ion beam
FKBP: FK506 binding protein
FRET: Förster resonance energy transfer
GABA: γ-aminobutyric acid
Grp75: glucose-regulated protein 75
H_2_O_2_: hydrogen peroxide
HRP: horseradish peroxidase
iLID: light-induced dimer
IMM: inner mitochondrial membranes
IP_3_: inositol 1,4,5-triphosphate
IP_3_: inositol triphosphate
IP_3_R: inositol triphosphate receptor
K^+^: Potassium ion
LDL: low-density lipoprotein
LE: late endosome
LiMETER: light-inducible membrane-tethered peripheral ER
LIT: light-induced tethering
LOCa: light-operated Ca^2+^ channel
LOV: light oxygen voltage-sensing
LTP: lipid transport proteins
MAM: mitochondria-associated membrane
MAPPER: membrane attached peripheral ER
MCSs: membrane contact sites
MCU: mitochondrial Ca^2+^ uniporter
MERLIN: mitochondria–ER length indicator nanosensor
Mfn2: mitofusin 2
miRISC: miRNA-induced silencing complex
M-L: mitochondria-lysosomal
MS: mass spectrometry
NIR: near-infrared
NRCAM: neuronal cell adhesion molecule
OMM: outer mitochondrial membranes
OptoID: photo-sensitive split TurboID
OSBP: oxysterol-binding protein
PA: phosphatidic acid
PALM: photoactivation localization microscopy
PB: polybasic
PCA: protein fragment complementation assay
PI: phosphatidylinositol
PI(3,5)P_2_: phosphatidylinositol 3,5-bisphosphate
PI3K: phosphatidylinositol 3-kinase
PLA: proximity ligation assay
PLC: phospholipids C
PM: plasma membrane
PS: Phosphatidylserine
*PTDSS1*: *Phosphatidylserine synthase 1*
PTPIP51: protein tyrosine phosphatase interacting protein 51
RyR: ryanodine receptor
SEM: scanning electron microscope
SF: subcellular fractionation
SIM: structured illumination microscopy
SMLM: single molecule localization microscopy
SOAR: STIM-ORAI activating region
SOCE: store-operated calcium entry
SP: signal peptide
SR: super-resolution
SR: super-resolution imaging
SREBP-2: sterol regulatory element-binding protein 2
STARD3: StAR-related lipid transfer domain-3
STARD3NL: STARD3 N-terminal like protein
STED: stimulated emission depletion microscopy
STORM: stochastic optical reconstruction microscopy
T2DM: type 2 diabetes
TG: Triglyceride
TGN: transGolgi network
TIRFM: total internal reflection fluorescence microscopy
TM: transmembrane
TPC: two-pore channel
TRP: transient receptor potential
TRPMLs: transient receptor potential mucolipin channels
UCNP: upconversion nanoparticles
VAPB: vesicle-associated membrane protein-associated protein B
VDAC: voltage-dependent anion selective channel
